# The XBB.1.5 mRNA booster vaccine does not significantly increase the percentage of XBB.1.5 mono-reactive T cells

**DOI:** 10.3389/fimmu.2025.1513175

**Published:** 2025-03-12

**Authors:** Joel Sop, Alicia Mercado, Alexis Figueroa, Tyler P. Beckey, Caroline C. Traut, Li Zhang, Kellie N. Smith, Joel N. Blankson

**Affiliations:** ^1^ Department of Medicine, Johns Hopkins Medicine, Baltimore, MD, United States; ^2^ Bloomberg-Kimmel Institute for Cancer Immunotherapy, Johns Hopkins Medicine, Baltimore, MD, United States; ^3^ Sidney Kimmel Comprehensive Cancer Center, Johns Hopkins University, Baltimore, MD, United States

**Keywords:** SARS-CoV-2, bivalent vaccine, T cell receptor, XBB.1.5, BA.2.86

## Abstract

Recent efforts in vaccine development have targeted spike proteins from evolving SARS-CoV-2 variants. In this study, we analyzed T cell responses to the XBB.1.5 and BA.2.86 subvariants in individuals who previously received bivalent vaccines containing mRNA for ancestral and BA.5 spike proteins. T cell-mediated cytokine responses to spike proteins from both variants were largely preserved. To determine the mechanism of this preserved recognition, we utilized the functional expansion of specific T cells (FEST) assay to distinguish between the presence of T cells that cross-recognized ancestral and variant epitopes versus distinct populations of T cells that were mono-reactive for ancestral or variant epitopes. We found the majority of spike-specific T cells cross-recognized the ancestral spike and the XBB.1.5 and BA.2.86 subvariants, with less than 10% of T cells being mono-reactive for either variant. Interestingly, immunization with the XBB.1.5 monovalent booster vaccine did not significantly increase the percentage of XBB.1.5 mono-reactive T cells. Our results suggest a potential limitation in the induction of mono-reactive T cell responses by variant-specific booster vaccines.

## Introduction

Understanding the dynamics of T cell responses to vaccines targeting specific SARS-CoV-2 variants is essential for shaping future vaccine strategies. Bivalent vaccines containing mRNA for both the ancestral and the Omicron BA.5 subvariant spike proteins were developed to improve humoral and cellular immunity against the BA.5 virus subvariant. However, these vaccines do not generate significantly better T cell and antibody responses to the Omicron BA.5 variant spike protein compared to monovalent ancestral spike mRNA vaccines (1–3). The Omicron XBB subvariants evolved from two Omicron BA.2 lineages and eventually replaced the BA.5 subvariant virus as the dominant virus in circulation (4). These new XBB subvariants were found to evade neutralizing antibody responses elicited by ancestral/BA.5 spike mRNA bivalent vaccines (4–11), and prompted the development of XBB.1.5 monovalent mRNA booster vaccines. The emergence of the BA.2.86 subvariant in 2023 marked another significant development in the landscape of SARS-CoV-2 variants. This subvariant has more than 30 mutations in the spike protein that differ from its precursor BA.2, and the BA.5 and XBB.1.5 variants (12) and is able to evade antibody responses elicited by natural infection and vaccination with ancestral and BA.5 spike mRNA (12–17).

In contrast to the evasion of antibody-mediated responses, vaccine-elicited T cells effectively recognize the XBB.1.5 and BA.2.86 subvariants (18–23). In a prior study, we demonstrated that ancestral/BA.5 bivalent mRNA vaccines mostly elicited cross-reactive T cell clonotypes that targeted conserved epitopes present in both spike proteins (24). In order to estimate the degree of protection that this vaccine would provide against a new highly mutated variant, we used the functional expansion of specific T cells (FEST) assay to better understand the degree of T cell cross-recognition of the BA.2.86 subvariant. This assay combines a peptide-stimulated T cell culture with sequencing of the T cell receptor (TCR) Vβ CDR3 region to identify antigen-specific TCRs (25–27). We have previously used it to analyze SARS-CoV-2 and endemic coronavirus spike-specific T cell responses (24, 28–30). XBB.1.5 monovalent booster mRNA vaccines boost the levels of neutralizing antibodies against a range of viral variants (31–40). However, less is known about its effect on T cell responses. We hypothesized that the lack of ancestral spike in this monovalent booster would lead to a better chance of production of XBB.1.5 mono-reactive T cells which is important as mono-reactive TCRs generally have higher functional avidity than cross-reactive TCRs (28). In this study, we used the FEST assay to analyze T cell responses to BA.2.86 spike peptides in recipients of the ancestral/BA.5 bivalent mRNA vaccine and responses to the XBB.1.5 variant before and the monovalent XBB.1.5 mRNA booster vaccine. Our results have implications for the development of vaccines for future SARS-CoV-2 variants.

## Methods

### Study participants

The study was approved by the IRB of Johns Hopkins University. Written informed consent was obtained from all participants prior to their inclusion in the study. The study population consisted of 5 participants who received either the Pfizer-BioNTech or Moderna SARS-CoV-2 ancestral spike/BA.5 spike bivalent vaccines and 6 participants who received the Pfizer-BioNTech or Moderna SARS-CoV-2 XBB.1.5 monovalent booster vaccines. 1 participant, HD58, was included in both studies. There were 5 men and 6 women with a median age of 33 (range 23-58 years of age). Details about the study participants are provided in **Supplementary Table 1**.

### ELISpot assay

An interferon-gamma ELISpot assay (Mabtech Science) was conducted using 181 overlapping ancestral spike peptides, varying in length from 12 to 17 amino acids with a 10 amino acid overlap, which were obtained from Biodefense and Emerging Infections (BEI) Research Resources. 125,000 to 200,000 PBMCs in 200 uL RPMI media with 10% fetal bovine serum were added to each well, and the plates were incubated for 20 hours with peptides at a concentration of 10 ug/ml. Two replicates per peptide were performed. ELISpot plates were analyzed by an independent investigator using an AID iSpot Spectrum and vendor-provided software to quantify IFN-y spot-forming units (SFUs) per well. SFU/million cells was calculated by adjusting spots per well with the appropriate dilution factor. The stimulation index (fold-change over untreated controls) for each donor was calculated by dividing the SFU of the peptide condition by the SFU of the untreated. A positive response was defined as a mean SFU of greater than or equal to 30 with a stimulation index of 3. All of the raw data for all the ELISpot assays are shown in **Supplementary Table 2**.

### Identification of antigen-specific TCRs using the FEST assay

We used the functional expansion of specific T cells (FEST) assay to identify mono- and cross-reactive TCRs. The FEST assay, which has been extensively reported, is a quantitative, reproducible assay that sequences the CDR3 region of the beta chain of the T cell receptor (TCR) of cells that have been cultured with peptide antigens, and therefore can identify expanded antigen-specific clones (25). Although it was originally developed to study cancer neoantigens (25–27), it has recently been adapted to robustly identify virus-specific T cell responses (24, 28–30, 41). The antigen specificity of TCRs identified via FEST has been previously validated by cloning the receptors into Jurkat cell lines (28), thus supporting the high accuracy of this assay in identifying bona fide antigen-specific TCRs. We have also used CMV, EBV and influenza peptides (JPT Peptide Technologies) as a control to confirm the specificity of the TCR response to SARS-CoV-2 spike peptides (28). For the present study, the ancestral, Omicron BA.5, and BA.2.86, spike peptide pools (JPT Peptide technologies) were utilized to activate CD8+ T cell-depleted PBMCs sourced from 6 participants. Initially, 2 million PBMCs were cultured in a medium comprising IMDM, 5% human AB serum, 10 IU/mL IL-2, and 50 μg/mL gentamicin, along with 1 μg/mL of either ancestral, BA.5, BA.2.86, or XBB.1.5 peptides. Each experimental condition underwent triplicate testing. Media replenishment occurred on days 3 and 7 by replacing half of it with fresh culture media. Harvesting of cells and subsequent DNA extraction from CD8+ T cell-depleted PBMCs utilized the QIAmp micro-DNA kit following the manufacturer’s guidelines (QIAGEN) on day 10. Subsequently, TCR-Seq analysis of DNA extracted from cultured CD8+ T cell-depleted PBMCs was conducted by the Johns Hopkins FEST and TCR Immunogenomics Core Facility using the AmpliSeq for Illumina TCR beta-SR panel. Unique molecular identifiers were used in the PCR amplification step to reduce amplification bias. Sequencing was performed on an Illumina MiSeq platform with unique dual indexes after pooling the samples. Preprocessing steps were implemented to remove nonproductive TCR sequences, align, and trim nucleotide sequences to retain only the CDR3 region. Sequences not conforming to specific criteria, such as those not starting with C or ending with F or W, and having fewer than 7 amino acids in the CDR3 were excluded. For CD8+ T cell-depleted PBMCs stimulated with BA.2.86 spike peptides, the median total number of receptors analyzed per person was 2,209 T cell receptors, ranging from 1,165 to 3,127 receptors. For CD8+ T cell-depleted PBMCs stimulated with XBB.1.5 spike peptide, the median total number of receptors analyzed per person was 2,148 T cell receptors and ranged from 1,593 to 3,070 receptors. Processed data files were then uploaded to our publicly available MANAFEST analysis web application (http://www.stat-apps.onc.jhmi.edu/FEST/) to bioinformatically identify antigen-specific T cell clonotypes. A response was considered positive based on specific criteria, including a mean frequency threshold of greater than 0.1% for each of the three replicates, with at least two replicates having a frequency greater than 0.1%, and the mean frequency being at least 5-fold greater than the mean frequency of wells containing DMSO alone. A mono-reactive response to ancestral, BA.5, BA.2.86, or XBB.1.5 spike was identified if all three conditions were met, and the mean frequency of the three replicates was also 5-fold higher than the response to the other spike protein. In the analysis of spike specific TCRs pre- and post-XBB.1.5 booster, we included TCRs that were identified as mono-reactive or cross-reactive TCRs pre-booster and were present post-booster, regardless of whether they expanded in the FEST assay post-booster. Additionally, we included spike specific TCRs that were identified post-booster and were also present pre-booster, irrespective of their expansion in the FEST assay pre-booster. All of the individual receptors analyzed in all the FEST assays are shown in **Supplementary Table 2**.

### Multiplex cytokine analysis with MSD assays

We conducted multiplex cytokine analysis using MSD assays, utilizing the V-Plex SARS-CoV-2 Panel 2 from MesoScale Diagnostics. This allowed us to measure TNF-alpha, IFN-gamma, and IL4 levels following stimulation with various spike peptides (ancestral, BA.5, BA.2.86, and XBB.1.5). Samples were collected from the cell culture medium on day 3 post-stimulation. Our assays followed the manufacturer’s protocol for accurate cytokine concentration measurement. Validation was performed by ensuring samples fell within the established detection range via standard curve and positive controls. Data analysis was conducted using the MSD discovery workbench software.

### Statistical analysis

Data were analyzed using GraphPad Prism (version 10.4.1). All statistical tests were two-tailed, and a p-value of < 0.05 was considered statistically significant.

**Figure 1:**
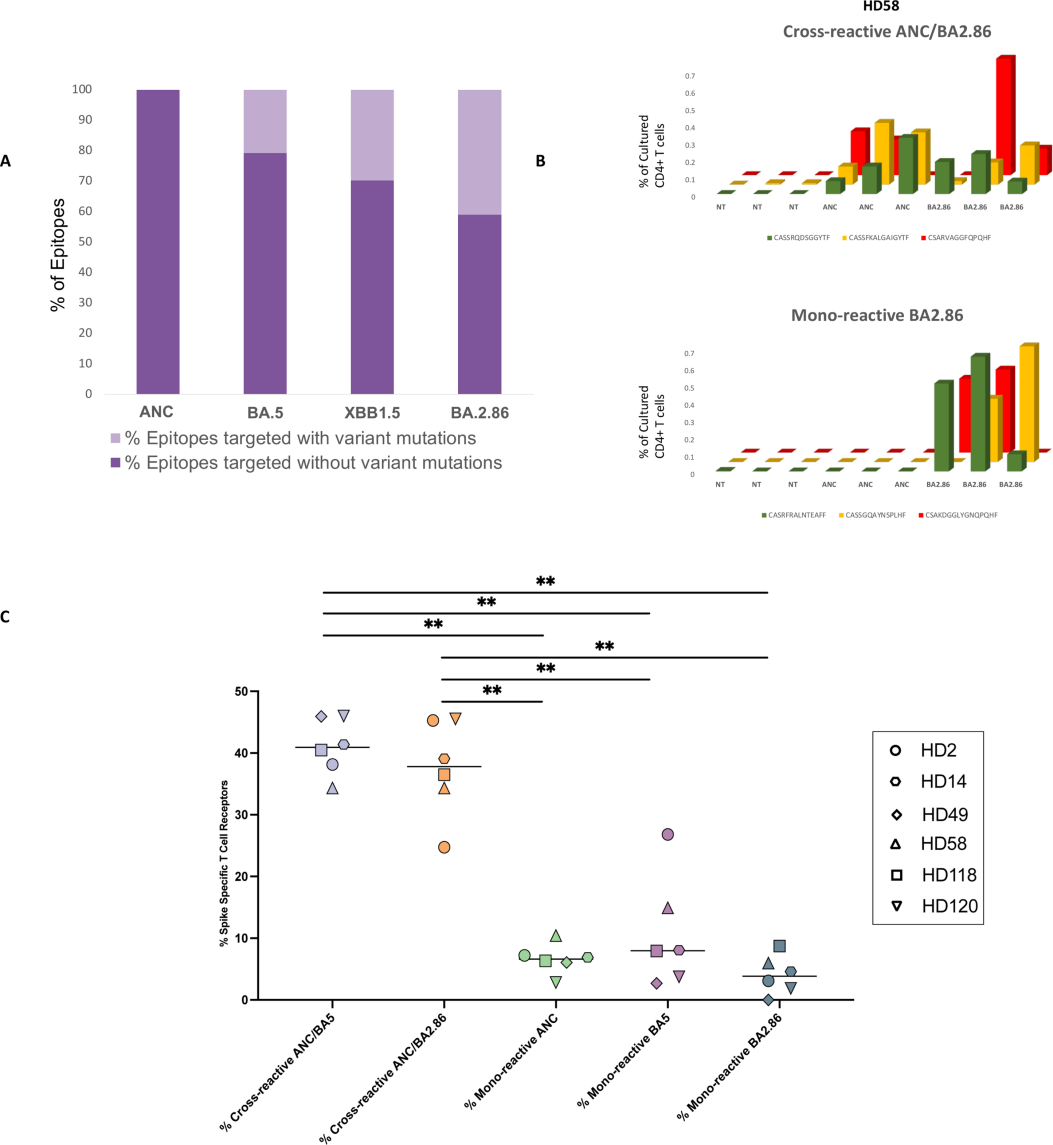
Analysis of mono-reactive and cross-reactive T cell receptors specific for ancestral and variant spike proteins prior to the receipt of the XBB.1.5 monovalent vaccine. **(A)** Overview of the percentage of T cell epitopes targeted across SARS-CoV-2 variants with and without mutations. **(B, C)** Antigen-specific TCR clonotypes were identified using the functional expansion of specific T cells (FEST) assay, a validated method that sequences the CDR3 region of the beta chain of the TCR to detect expanded antigen-specific clones. The FEST assay distinguishes between cross-reactive and mono-reactive TCRs by assessing whether the same clonotype expands in response to multiple spike variants or only one. Cross-reactivity was defined by the functional expansion of the same CD4+ TCR clonotypes in response to pooled ancestral (ANC), Omicron BA.5 (BA.5), and Omicron BA.2.86 (BA.2.86) peptides. Mono-reactivity was defined as expansion to only 1 of the 3 spike peptide pools. Three technical replicates were performed for each peptide culture in each of the 6 participants. A representative mono-reactive and a representative cross-reactive TCR β clonotypes are shown for a participant (HD58) **(B)**. Data are shown as the (%) frequency after culture (y axis) of antigen-specific CD4+ T cell clonotypes (z axis) for the peptide pools tested (x axis). **(C)** The percentage of spike-specific TCRs that were cross-reactive versus mono-reactive for all 6 participants are shown. ∗∗p < 0.01. The FEST assay allows for quantitative differentiation of cross-reactive and mono-reactive TCRs by analyzing TCR expansion following antigen stimulation. The median value for each group is indicated by the horizontal line within each set of data points. Statistical significance was determined using the One-Way ANOVA with Tukey Multiple Comparison Testing. ∗∗p < 0.01.

For TCR and cytokine analysis where multiple conditions were compared across the same set of participants at a single time point (**Figure 1C**, **Supplementary Figure 2**), One-Way ANOVA with Tukey Multiple Comparison Testing was performed.

**Figure 2:**
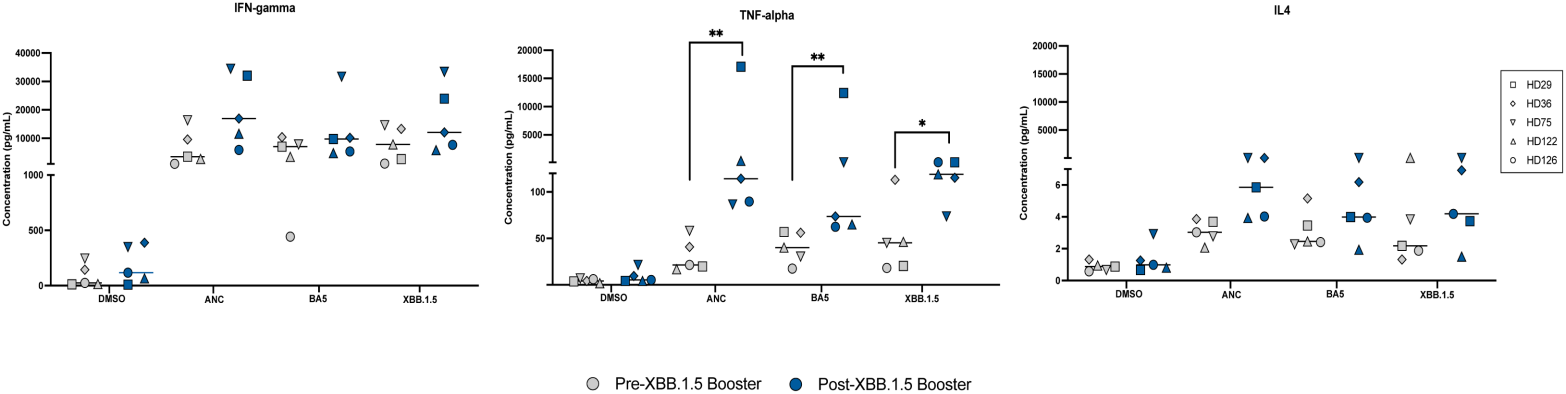
Cytokine Responses to Ancestral, BA.5, and XBB.1.5 Spike Peptides Pre and Post XBB.1.5 Booster Administration. Cytokine levels of IFN-gamma, TNF-alpha, and IL4 were measured in pg/mL using an MSD ELISA assay following stimulation with different spike peptides before and after XBB.1.5 booster administration. This figure displays the cytokine responses elicited by stimulation with the three spike proteins both pre and post booster administration. Symbols in gray represent measurements before the booster, and symbols in blue represent measurements after the booster. The median value for each group is indicated by the horizontal line within each set of data points. Statistical significance between pre- and post-vaccine measurements was assessed using the paired t-test. *p<0.05; **p < 0.01.

**Figure 3:**
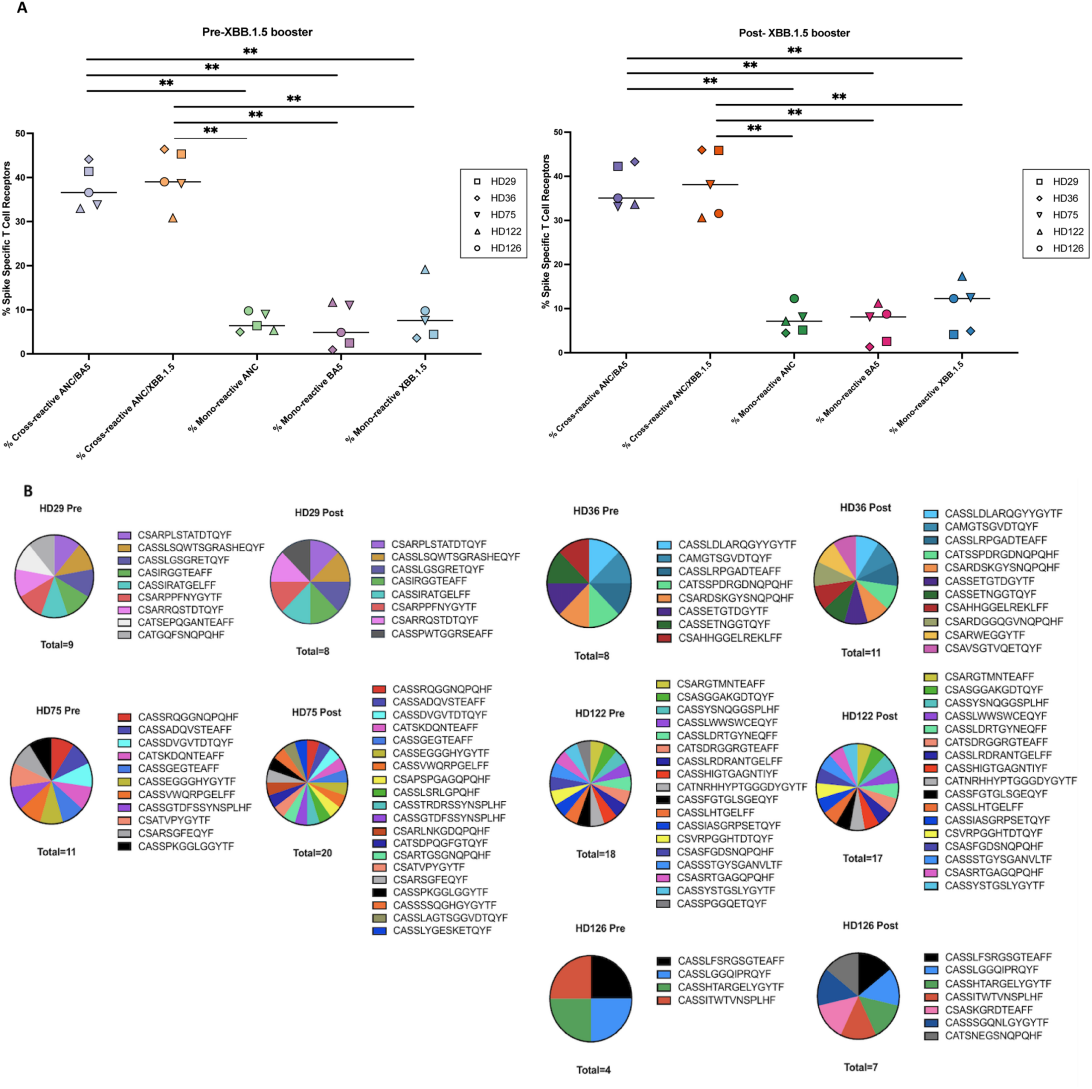
Mono-reactive and cross-reactive T cell receptors and targeted spike peptides before and after the XBB.1.5 spike vaccine. **(A)** Antigen-specific TCR clonotypes were identified using the functional expansion of specific T cells (FEST) assay, a validated method that sequences the CDR3 region of the beta chain of the TCR to detect expanded antigen-specific clones. The FEST assay distinguishes between cross-reactive and mono-reactive TCRs by assessing whether the same clonotype expands in response to multiple spike variants or only one. Cross-reactivity was defined by the functional expansion of the same CD4+ TCR clonotypes in response to pooled ancestral (ANC), Omicron BA.5 (BA.5), and XBB.1.5 peptides. Mono-reactivity was defined as expansion to only 1 of the 3 spike peptide pools. Three technical replicates were performed for each peptide culture in each of 5 participants before and after booster administration. The FEST assay allows for quantitative differentiation of cross-reactive and mono-reactive TCRs by analyzing TCR expansion following antigen stimulation. The median value for each group is indicated by the horizontal line within each set of data points. Statistical significance was determined using the One-Way ANOVA with Tukey Multiple Comparison Testing. ∗∗p < 0.01. **(B)** Pie charts depicting the distribution of individual T cell receptor (TCR) clonotypes specific to the XBB.1.5 spike protein among study participants before (Pre-Booster) and after (Post-Booster) XBB.1.5 booster administration. Each pie chart represents the proportion of mono-reactive TCRs targeting the XBB.1.5 spike protein within the total spike-specific T cell population for each participant.

The paired t-test was used to compare cytokines and the frequency of XBB.1.5 mono-reactive TCRs before and after the XBB.1.5 monovalent vaccine (**Figures 2**, **3**).

**Figure 4:**
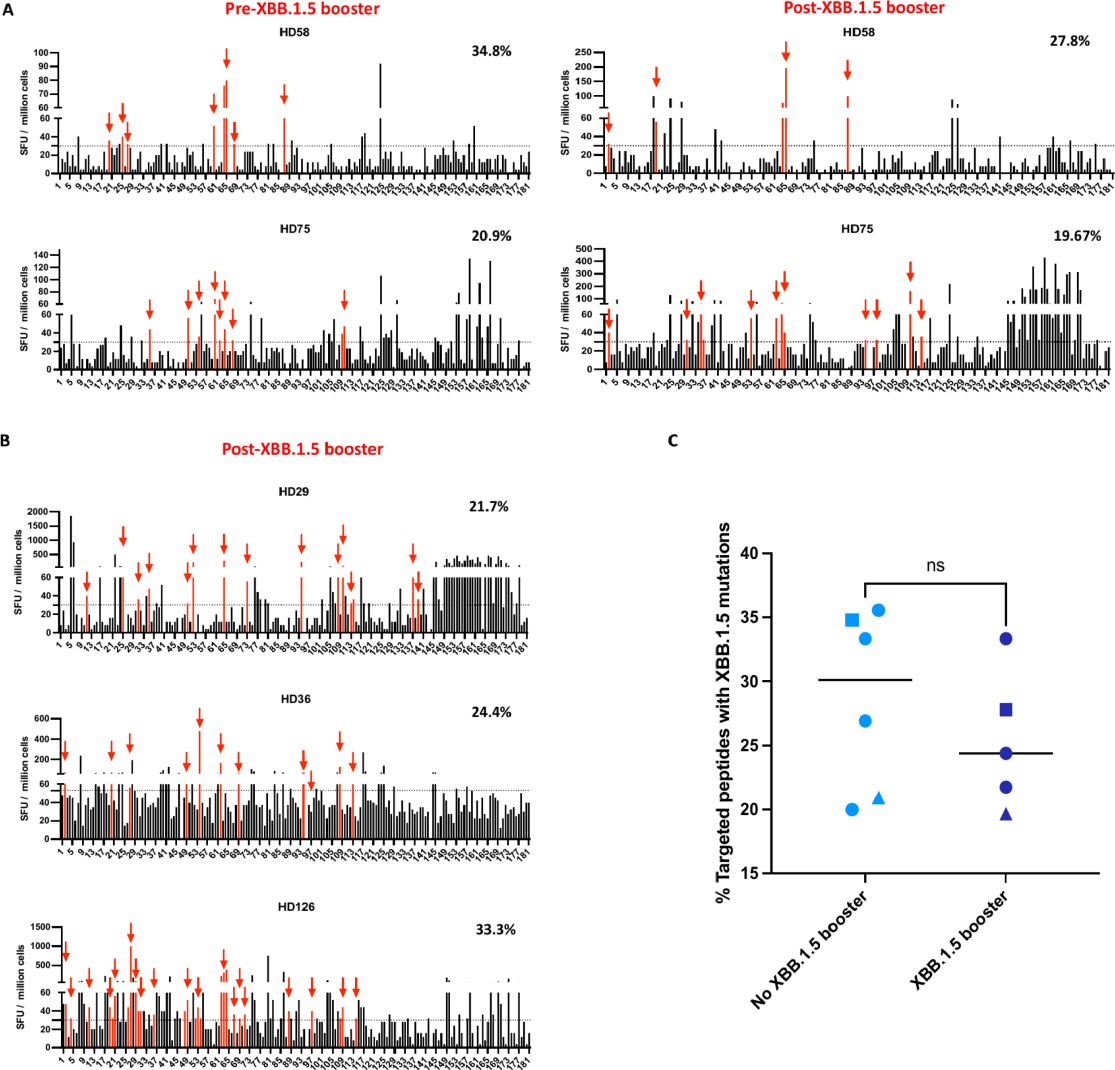
The percentage of ancestral spike peptides targeted by T cells that contain XBB.1.5 mutations pre and post the XBB.1.5 booster. **(A)** The IFN-γ ELISpot assay was performed on samples obtained from 2 participants before and after XBB.1.5 booster administration. The SFU of PBMCs in response to each of the 181 overlapping peptides are shown. Each data point represents the mean of 2 technical replicate values. The dotted horizontal line represents the threshold for positive responses (SFU ≥30). The red vertical bars represent targeted peptides that would contain an XBB.1.5 mutation. The percentages on the right represent the percentage of targeted peptides that would contain an XBB.1.5 mutation. **(B)** ELISpot assay performed on samples from 3 participants after XBB.1.5 booster administration. **(C)** Comparison of percentage of peptides targeted by T cells that contain XBB.1.5 mutations in participants who either received or did not receive the XBB.1.5 booster. The median value for each group is indicated by the horizontal line within each set of data points. Statistical significance between the two groups was assessed using the Mann-Whitney U test for unpaired samples. ns, not significant. The squares represent HD58, and the triangles represent HD75. The circles represent different participants, with each circle corresponding to a unique individual.

For the comparison of epitopes in study participants who did and did not receive the XBB.1.5 booster (**Figure 4C**), the Mann-Whitney U Test was performed.

## Results

### Higher degree of mutations in targeted T cell epitopes in XBB.1.5 and BA.2.86 subvariants

We first tested the individual response to 181 overlapping peptides spanning the ancestral spike protein using IFNγ ELISpot (24) in 6 healthy donors who had received the ancestral and BA.5 spike mRNA bivalent vaccine. A median of 32 epitopes per donor was identified (range 5 to 90). Of these epitopes, a median of 20.8%, 29.9%, and 40% contained amino acid mutations in the BA.5 (24), XBB.1.5 and BA.2.86 subvariants respectively (**Figure 1A**, **Supplementary Figure 1**).

### Comparable cytokine responses to ancestral, BA.5 and BA.2.86 spike peptides in bivalent vaccine recipients

We implemented a multiplex electrochemiluminescent assay to measure the secretion of IFN-gamma, TNF-alpha, and IL-4 following stimulation with different spike peptides. There were no significant differences in the secretion of any of the cytokines measured in response to stimulation with ancestral peptides versus BA.5 and BA.286 peptides (**Supplementary Figure 2**).

### High degree of T cell cross-recognition of ancestral and subvariant spike peptides

Using the FEST assay, we assessed the proportion of CD4+ T cells demonstrating cross-recognition of ancestral BA.5 or BA.2.86 spike proteins in 6 donors. We also identified the percentage of clonotypes that were mono-reactive for each spike protein. After culturing CD8+ T cell-depleted PBMCs with ancestral, BA.5, or BA.2.86 spike peptide pools, we sequenced T cell receptor (TCR) Vβ CDR3 repertoires on day 10. We then used a bioinformatic pipeline to identify mono- and cross-reactive TCR clonotypic expansions (26). Across our participant cohort, we observed 5 broad categories of spike-specific TCRs: those mono-reactive to ancestral, BA.5, or BA.2.86 peptides; those cross-reactive for ancestral and BA.5 peptides and those cross-reactive for ancestral and BA.2.86 spike proteins. Examples of cross-reactive and mono-reactive clonotypes are shown in **Figure 1B**. For each of the six participants analyzed, we determined the absolute number of TCRs falling into each category and then calculated the percentage of spike-specific TCRs exhibiting mono-reactivity versus cross-reactivity.

As shown in **Figure 1C**, the median percentage of total spike-specific T cells demonstrating cross-reactivity to ancestral and BA.5 spike protein was 40.9% (range: 34.3%-46.0%). The median percentage of T cells that cross-recognized ancestral and BA.2.86 spike peptides was comparable at 37.8% (range: 24.7%-45.5%). 6.6% of the spike-specific T cells were mono-reactive for the ancestral spike protein (range: 2.8%-10.4%), while 8% (range: 2.8%-26.8%) and 3.8% (range: 0.0%-8.7%), of spike-specific T cells were mono-reactive for the BA.5 and BA.2.86 subvariants respectively.

### Cytokine responses to ancestral, BA.5 and XBB.1.5 spike peptides pre and post the XBB.1.5 booster

We measured T cell responses in 5 individuals before and after the XBB.1.5 booster. IFN-gamma, TNF-alpha, and IL4 concentrations were measured in response to stimulation of CD8+ T cell-depleted PBMCs with different spike peptides pre- and post-XBB.1.5 booster administration. The median concentrations of IFN-gamma and IL4 were not significantly different in response to stimulation with the different spike peptides pre and post the XBB.1.5 booster. In contrast, there was a significant increase in TNF-alpha secretion after stimulation with all 3 spike proteins after the booster was given (**Figure 2**).

### The XBB.1.5 booster does not increase the percentage of T cell clonotypes that are mono-reactive for the XBB.1.5 spike protein

Mono-reactive TCRs have previously been shown to have higher functional avidity than cross-reactive TCRs (28, 42). Thus, we used the FEST assay to assess the proportion of CD4+ T cells that were cross-reactive for ancestral and BA.5, or ancestral and XBB.1.5 spike proteins compared to those that were mono-reactive for each protein before and after the XBB.1.5 booster. As shown in **Figure 3A**, the median percentage of total spike-specific T cells that were cross-reactive for ancestral and BA.5 spike protein was 36.6% (range: 33.0%-44.1%) before the XBB.1.5 booster, and 35.1% (range: 33.1%-43.3%) after the booster. The percentage of TCRs that were cross-reactive for ancestral and XBB.1.5 spike protein was 39.0% (range: 30.9%-46.4%) pre-booster and 38.1% (range: 30.6%-46.0%) after the booster. The percentage of cells that were mono-reactive for the ancestral spike protein was 6.4% (range: 5.0%-9.8%) pre-booster and 7.1% (range: 4.5%-12.3%) after the booster. A median of 4.9% of the cells were mono-reactive for BA.5 spike protein (range: 0.9%-11.7%) pre-booster, with a median of 8.1% (range: 1.3%-11.2%) after the booster. Most importantly, a median of 7.6% of the cells were mono-reactive for XBB.1.5 spike (range: 3.6%-19.1%) before the booster, compared to a median of 12.3% (range: 4.1%-17.3%) after the booster. There were also similar numbers of individual clonotypes mono-reactive for XBB.1.5 peptides before and after the booster (**Figure 3B**).

### The percent of epitopes with potential XBB.1.5 mutations targeted by T cells did not significantly change after the XBB booster

We next asked whether the XBB.1.5 booster would lead to an increased number of targeted peptides that would be affected by XBB.1.5 mutations. PBMCs from 2 study participants (HD58 and HD75) were stimulated with each of 181 overlapping ancestral spike peptides individually in an IFN-gamma ELISpot. In these participants, 23 and 43 individual peptides respectively were identified as targets before the booster. Of these targeted peptides, 34.8% and 20.9% would be affected bymutations in the XBB.1.5 subvariant. After the booster, 18 and 61 individual peptides were targeted (**Figure 4A**). The percentage of these peptides that have mutations in the XBB.1.5 subvariant was not markedly different (27.8% and 19.7% respectively, **Figure 4A**). We also analyzed another 3 individuals only after the XBB.1.5 booster because PBMCs before the booster were not available. These individuals targeted 21.7%, 24.4%, and 33.3% of peptides respectively that would be affected by XBB.1.5 mutations (**Figure 4B**). In all, the 5 recipients of the XBB.1.5 booster did not target significantly more of the ancestral spike peptides with potential XBB.1.5 mutations than 6 participants who either did not receive this booster or were tested before the booster was given (**Figure 4C**).

## Discussion

We have previously used the FEST assay to distinguish between mono-reactive and cross-reactive TCRs in a study of T cell responses to spike peptides from common cold coronaviruses and SARS-CoV-2 (28). In order to validate our finding, we cloned the alpha and beta genes of 8 TCRs we identified as being mono-reactive and 5 TCRs we identified as cross-reactive and transfected them into a CD4-overexpressing Jurkat NFAT luciferase reporter system. Transfected cells expressing TCRs that we identified as being cross-reactive responded to spike peptides from SARS-CoV-2 and the common cold coronavirus, HCoV-NL63, whereas cells with TCRs identified as being mono-reactive only responded to SARS-CoV-2 spike peptides (28), thus supporting the notion that the FEST assay can reliably distinguish between mono-reactive and cross-reactive TCRs.

In a subsequent study with this assay, we demonstrated that less than 1% of the SARS-CoV-2 specific clonotypes elicited by monovalent vaccines with ancestral spike mRNA were cross-reactive with common cold coronavirus spike peptides (29). In contrast, we recently showed that up to 80% of the spike-specific T cell clonotypes that were elicited by ancestral/BA.5 bivalent mRNA vaccines cross-recognized the ancestral and BA.5 spike peptides (24). Our epitope analysis suggested that this phenomenon may have been mainly due to the fact that T cells targeted epitopes that were identical in the ancestral and BA.5 spike proteins.

The BA.2.86 subvariant contains more than 30 distinct spike mutations compared to the BA.5 subvariant. A higher percentage of these mutations are in targeted T cell epitopes which could potentially lead to immune escape. Thus, we expected to see lower levels of T cell cross-recognition of this subvariant in recipients of ancestral/BA.5 bivalent mRNA vaccines. Instead, we found very similar frequencies of T cell clonotypes that cross-recognized ancestral and BA-5 spike peptides versus ancestral and BA.2.86 spike peptides. We also saw similar levels of cytokine secretion by T cells in response to peptides from the ancestral, BA.5, and BA.2.86 spike proteins which is further proof of T cell cross-recognition of these proteins. This continued recognition of a variant with 30 mutations is reassuring and can potentially be explained by the breadth of the T cell response following immunization with the monovalent ancestral spike vaccine (24, 43).

We previously showed that ancestral/BA.5 spike bivalent mRNA vaccines do not elicit a high frequency of BA.5 mono-reactive T cells. We hypothesized that the XBB.1.5 spike monovalent booster would induce higher levels of variant-specific T cells as in the absence of the ancestral spike protein, T cells would be more likely to respond to epitopes containing XBB.1.5 mutations. Interestingly, the levels of XBB.1.5 mono-reactive T cells after vaccination were similar to the levels of BA.5 mono-reactive T cells after the bivalent mRNA vaccine. This finding could be because 70% of epitopes targeted by individuals are not mutated in the XBB.1.5 subvariant. Indeed, we did not observe a shift toward recognition of more epitopes that are mutated in the XBB.1.5 variant in individuals who received the XBB.1.5 booster, including HD58 and HD75 who we studied before and after vaccination. Interestingly, while we did not observe an overall difference in the frequency of mono-reactive TCRs in our cohort of 5 individuals, we did observe an increase in the number of HD75 TCR clonotypes that were mono-reactive for XBB.1.5 spike peptides after the monovalent vaccine. The difference in the TCR and ELISpot responses in this individual can potentially be explained by the fact that the ELISpot assay detects cytokine responses that are distinct from proliferative responses that impact the TCR clonotypes measured in the FEST assay. The fact that higher levels of TNF were secreted in responses to stimulation with spike peptides does suggest that the XBB.1.5 vaccine may have had a qualitative effect on spike-specific CD4+ T cells which needs to be further explored.

Our work is limited by the fact that we did not analyze vaccine-induced spike-specific CD8+ T cell responses, despite their demonstrated role in protective immunity (44) due to the limited number of cells available and the fact that we do not observe a strong expansion of SARS-CoV-2-specific memory CD8+ T cells in many individuals. We also studied a relatively small number of healthy donors, which may limit the generalizability of our findings. Furthermore, due to our limited sample size, we were unable to conduct subgroup analyses based on factors such as age, gender, and prior SARS-CoV-2 infection history. Finally, due to the large numbers of peptides that were targeted, we were not able to determine the optimal epitope in each peptide, which may impact precise mapping of immunodominant T cell targets. However, the use of technical replicates and the large number of T cell receptors analyzed helped improve the robustness of the dataset and our results are consistent with other studies that analyzed functional responses to ancestral and XBB.1.5 spike proteins after the XBB.1.5 booster vaccine (45, 46) or after natural infection with this variant (47). Future studies with larger cohorts are needed to confirm our TCR analysis and epitope mapping findings.

In summary, our data suggest that current mRNA vaccines induce high levels of spike-specific CD4+ T cells that can cross-recognize emerging variants. However, there does not appear to be an effective induction of mono-reactive T cell responses even with variant-specific booster vaccines. Antigenic imprinting has been observed in spike-specific B cells after the XBB.1.5 monovalent vaccine (48, 49). A study suggests that repeated exposure to Omicron spike proteins can overcome this process (50). While antibodies mainly target the receptor binding and N terminal domains of spike, T cells recognize many epitopes throughout the entire protein, the majority of which are identical in ancestral and variant spike proteins. Thus, antigenic imprinting would be less likely to impact the total T cell response. Nonetheless, it would be interesting to determine whether repeated exposure to variant spike proteins would also improve the mono-reactive T cell response. Our study suggests that variant-specific vaccines may not improve T cell responses unless the targeted variants have a high frequency of mutations in pre-existing T cell epitopes.

## Data Availability

The original contributions presented in the study are included in the article/**Supplementary Material**, further inquiries can be directed to the corresponding author/s.
