# Supplementary material for: The XBB.1.5 mRNA booster vaccine does not significantly increase the percentage of XBB.1.5 mono-reactive T cells
Source: Front Immunol. 2025 Mar 12;16:1513175. doi: 10.3389/fimmu.2025.1513175 (PMC11936820; doi:10.3389/fimmu.2025.1513175)
Supplement: Supplementary file 1 [file DataSheet1.pdf]

Supplementary figures legend

Supplementary Figure 1: T cell responses to individual overlapping ancestral spike peptides.

The IFN-gamma ELISpot assay was performed on samples obtained from XBB.1.5 booster recipients. The SFU of PBMCs in response to each of the 181 overlapping peptides are shown. Each data point represents the mean of 2 technical replicate values (n = 8 for XBB.1.5 and n=8 for BA.2.86/JN.1). The dotted horizontal line represents the threshold for positive responses. The red and blue vertical bars represent targeted peptides that would contain an XBB.1.5 and BA.2.86/JN.1 mutation respectively. The percentages on the right represent the percentage of targeted peptides that would contain a XBB.1.5 or BA.2.86/JN.1 mutation.

Supplementary Figure 2: Cytokine responses to Ancestral, BA.5, and BA2.86 Spike peptides in bivalent vaccine Recipients.

Cytokine levels of IFN-gamma, TNF-alpha, and IL4 were measured in pg/mL using an MSD ELISA assay following stimulation with different spike peptides.

Supplemental figure 1

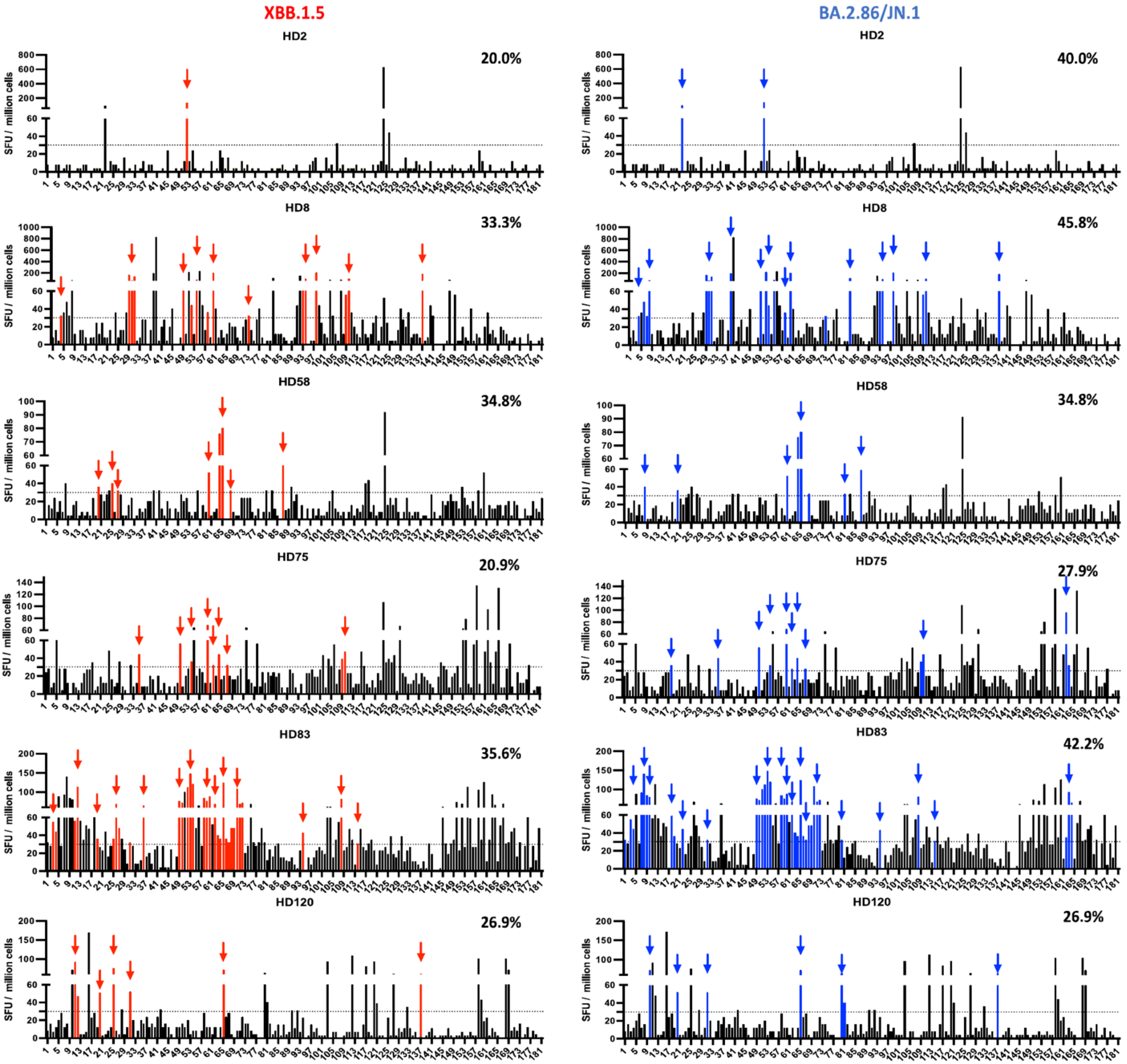

Supplemental figure 2

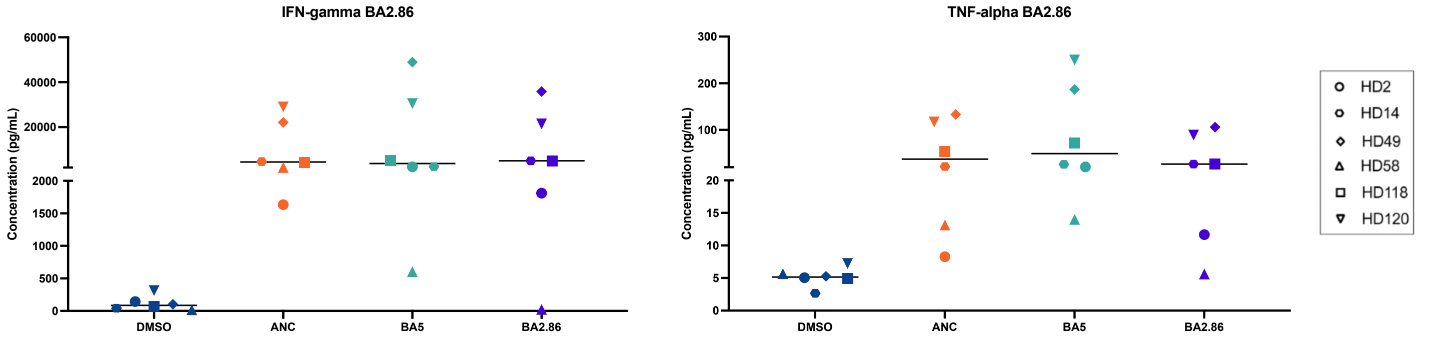

Supplementary Table 1. Study participant demographic data

Participant	Time between vaccine and blood draw (days)	Vaccine	Prior positive COVID test	Biological sex	Age
HD2	490	Ancestral/BA.5	-	Male	47
HD14	486	Ancestral/BA.5	+	Female	33
HD49	474	Ancestral/BA.5	+	Female	28
HD58	465	Ancestral/BA.5	+	Male	29
HD118	435	Ancestral/BA.5	+	Female	33
HD120	394	Ancestral/BA.5	+	Female	23
HD29	0, 42	XBB.1.5	-	Female	58
HD36	-5, 32	XBB.1.5	+	Male	45
HD58	-153, 21	XBB 1.5	+	Male	29
HD75	-5, 31	XBB.1.5	+	Male	28
HD122	-1, 78	XBB.1.5	+	Male	35
HD126	0, 58	XBB.1.5	+	Female	54

The recipients of the XBB.1.5 vaccine received the vaccine a median of 394 days after the ancestral/BA.5 spike mRNA vaccine (range of 372-458 days).
